# Efficacy of a nanoparticle vaccine administered *in-ovo* against *Salmonella* in broilers

**DOI:** 10.1371/journal.pone.0247938

**Published:** 2021-04-06

**Authors:** Keila Acevedo-Villanueva, Sankar Renu, Renukaradhya Gourapura, Ramesh Selvaraj

**Affiliations:** 1 Department of Poultry Science, The University of Georgia, Athens, GA, United States of America; 2 Food Animal Health Research Program, Ohio Agricultural Research and Development Center, The Ohio State University, Wooster, OH, United States of America; Tokat Gaziosmanpasa Universitesi, TURKEY

## Abstract

*Salmonella* is a zoonotic pathogen that persists in poultry. *Salmonella* vaccines that can be delivered *in-ovo* can be cost-effective and can decrease *Salmonella* load in poultry. This study evaluates the efficacy of a *Salmonella* chitosan-nanoparticle (CNP) vaccine, administered *in-ovo*, in broilers. CNP vaccine was synthesized with *Salmonella* Enteritidis (SE) outer-membrane-proteins (OMPs) and flagellin proteins. At embryonic-d18, one-hundred-thirty-six eggs were injected with 200μl PBS or 1000μg CNP into the amniotic cavity. At d1-of-age, 132 chicks were allocated in 6 pens/treatment with 11 chicks/pen. At d7, birds were orally challenged with 1×10^9^ CFU/bird SE. At d1, 8h-post-challenge, d14, and d21, serum anti-SE-OMPs IgY were analyzed. At d14 and d21, cloacal swabs and bile anti-SE-OMPs IgA, CD4^+^/CD8^+^-T-cell ratios, and ceca SE loads were analyzed. At d21, cecal tonsil IL-1β, IL-10, and iNOS mRNA were analyzed. Body-weight-gain (BWG) and feed-conversion-ratio (FCR) were recorded weekly. Data were analyzed by Student’s t-test at P<0.05. There were no significant differences in BWG or FCR between vaccinated birds compared to control. At d1, CNP-vaccinated birds had 5.62% greater levels (P<0.05) of anti-SE-OMPs IgY, compared to control. At 8h-post-challenge, CNP-vaccinated birds had 6.39% greater levels (P<0.05) of anti-SE-OMPs IgY, compared to control. At 2wk-post-challenge, CNP-vaccinated birds had 7.34% lower levels (P<0.05) of anti-SE-OMPs IgY, compared to control. At 1wk-post-challenge, CNP-vaccinated birds had 15.30% greater levels (P<0.05) of bile anti-SE-OMPs IgA, compared to control. At d14 and d21, CNP-vaccinated birds had 0.62 and 0.85 Log10 CFU/g, decreased SE ceca load (P<0.05), respectively, compared to control. There were no significant differences in CD4^+^/CD8^+^-T-cell ratios between vaccinated birds compared to control. There were no significant differences in IL-1β, IL-10, iNOS mRNA between vaccinated birds compared to control. Findings demonstrate that the *in-ovo* administration of CNP vaccine can induce an antigen-specific immune response against SE and can decrease SE cecal load in broilers.

## Introduction

*Salmonella* is an enteric pathogen in poultry. Birds can have up to Log 5 of *Salmonella* CFU and remain asymptomatic [1]. In humans, more than 70% of salmonellosis cases in the United States have been attributed to the consumption of contaminated poultry products or eggs [2]. The Centers for Disease Control and Prevention estimates that *Salmonella* causes about 1.35 million infections, 26,500 hospitalizations, and 420 deaths in the United States every year [3].

To prevent *Salmonella* infections and the economic burden it brings, the poultry industry employs the use of *Salmonella* vaccines, but currently, commercial vaccines have certain disadvantages. Live vaccines are not preferred due to the ability of the live strain to regain its virulence and spread through shedding, interfere with the salmonellosis monitoring program [4], and strict cold chain requirement to preserve their function. Killed vaccines are preferred, but the intramuscular route of administration is time-consuming, increases tissue damage, and requires additional vaccine administration costs. An oral-killed vaccine would circumvent the disadvantages of both live and killed vaccines while mimicking a natural infection and stimulating the mucosal and systemic immune responses [5]. However, there are currently no commercially available oral-killed *Salmonella* vaccines for poultry due to the acidic pH of the gastrointestinal tract (GIT) [6].

Nanoparticle vaccines are good carriers to deliver antigens through the oral route because the polymer coating that surrounds the vaccine antigens aids in preventing the degradation of the proteins of interest [7–9]. Chitosan nanoparticles intrinsically possess a positive surface charge that facilitates their adherence to the negatively charged mucus layers, ensuring delivery of the vaccine antigens [10, 11]. Also, nanoparticles can provide a slow release of the antigen within the GIT, which gives a constant stimulus to mucosal immune cells that can result in reducing the dosing frequency or the need for adjuvants [7]. Our polymeric nanoparticle vaccines, against *Salmonella*, have been previously characterized and used for antigen delivery in poultry [12–14]. Findings have shown that both, chitosan and polyanhydride, *Salmonella*-nanoparticle vaccines are safe with no adverse effects detected on bird performance or health and can significantly increase an antigen-specific humoral and mucosal immune response, as well as decrease the *Salmonella* load in the ceca.

It is important to have effective methods of mass vaccination because flock sizes of commercial poultry contain thousands of birds. *In-ovo* vaccines are a mass vaccination strategy that can reduce stressful post-hatching procedures [15]. *In-ovo* vaccines are currently approved for use in poultry flocks for different diseases, e.g., Marek’s disease. Typically, the *in-ovo* vaccine is injected into the amniotic sac and the amnion fluid containing the vaccine antigen gets swallowed by the embryo, giving way to “oral vaccination” at an early stage [16]. *In-ovo* vaccination induces early immunity with less interference from maternal antibodies [15]. Early immunity can also aid in significantly reducing the inflammatory response upon future encounters with the antigen [17]. Successful vaccination against *Salmonella* at an embryonic stage would be a useful prevention strategy for the poultry industry.

A *Salmonella* CNP vaccine was synthesized with crude-enriched outer membrane proteins (OMPs) and flagellin protein extracts from *Salmonella* Enteritidis (SE) and surface-tagged with flagellin proteins [12]. Previous findings demonstrate that the 1000μg CNP *Salmonella* vaccine can induce a specific immune response against *Salmonella* and has the potential to mitigate SE cecal colonization in broiler birds [13]. The objective of this study is to evaluate the efficacy of a novel chitosan nanoparticle oral vaccine (CNP) administered *in-ovo* for *Salmonella* control in broilers. We hypothesize that the *in-ovo* administration of the *Salmonella* CNP vaccine will increase anti-*Salmonella* antigen-specific IgY and IgA and decrease the SE loads in broilers.

## Materials and methods

### Isolation of outer membrane proteins

Outer membrane proteins from SE were isolated as described previously [12] with few modifications. Briefly, a bacterial culture, at its stationary phase, was washed with 10 mM Tris buffer (pH 7.5), the sediment was suspended in Tris-sucrose EDTA (pH 8), and incubated on ice for 90 min. The cell suspension was centrifuged at 16,000 ×g for 30 min and the supernatant was collected and centrifuged at 100,000 ×g for 60 min. The pellet containing OMPs enriched extract was freeze-dried with 5% sucrose as a cryoprotectant. The protein concentration was estimated using a micro-BCA protein assay kit (Thermo Scientific, MA) as per the manufacturer’s instructions.

### Isolation of flagellin proteins

Flagellin proteins from SE were isolated as described previously [12]. *Salmonella* Enteritidis bacterial culture was grown on Trypticase soy agar plates and was inoculated into brain heart infusion broth and subsequently incubated for 48 h at 37°C, without shaking. The cells were washed with PBS (pH 7.4) and centrifuged at 7000 ×g for 30 min. The cell pellet was treated with 3M potassium thiocyanate (Sigma, MO) in PBS for 2 h at room temperature, under magnetic stirring. The cell suspension was centrifuged at 35,000 ×g for 30 min and the supernatant containing flagellin-protein-enriched extract was dialyzed once against PBS (pH 7.4), followed by Milli-Q water, and freeze-dried with 5% sucrose as a cryoprotectant. The protein concentration was estimated using micro-BCA protein assay kit as per the manufacturer’s instructions.

### Preparation of nanoparticle vaccine

The CNP vaccine was synthesized at the Food Animal Health Research Program, The Ohio State University, USA. The OMPs and flagellin proteins were isolated from SE and the CNP vaccine was synthesized using the ionic gelation method, as described previously [12]. Briefly, a solution of 1.0% (w/v) low molecular weight chitosan (Sigma, MO) was prepared by slowly dissolving chitosan in an aqueous solution of 4.0% acetic acid. The solution was sonicated, adjusted to a 4.3 pH, and was filtered using a 0.44 μm syringe filter. Five milliliters of the 1.0% chitosan solution was added to 5 mL of dH2O and incubated with 2.5 mg OMPs and flagellar proteins. To form the nanoparticles, 2.5 mL of 1% (w/v) TPP in 2.5 mL deionized water was added to the above solution under magnetic stirring at room temperature. Afterward, 2.5 mg of flagellin protein in PBS was added to the nanoparticles to surface-conjugate the nanoparticles with flagellin proteins. The CNP vaccines were collected by centrifugation at 10,500 ×g for 10 minutes, lyophilized, and stored at -80°C until further use.

### Experimental animals

All animal protocols were approved by the Institutional Animal Care and Use Committee at the University of Georgia. Birds (Cobb-Vantress hatchery, Inc. Cleveland, GA, USA) had access to *ad libitum* feed and water. Birds were monitored at least once a day for dehydration, refusal to eat food, loss of body weight, diarrhea, bloody feces, and lethargy during the experimental period. All birds were euthanized by cervical dislocation. All birds were terminated at the end of the experimental period.

A total of one hundred thirty-six broilers eggs were vaccinated at embryonic day 18 of incubation. The eggs were randomly assigned to two treatment groups: control (sterile phosphate-buffered saline; PBS) and immunized (CNP). The eggshell was disinfected by spraying 1.5% hydrogen peroxide and was punctured using a sterile needle. Two hundred microliters of either PBS or 1000 μg vaccine in 200 μl PBS were injected into the amniotic cavity as described earlier [18]. The vaccinated eggs were incubated under standard hatchery settings. All the eggs were vaccinated and transferred from the incubator to the hatcher at embryonic day 18. At 21d of incubation, the percentage of hatchability was recorded. The hatchability was 97%.

At d1 of age, one hundred thirty-two chicks were randomly allocated to the two treatment groups in six pens (n = 6) with 11 chicks/pen. At d1, d7, d14, and d21 of age, body weight and feed consumption were recorded, and body weight gain (BWG) and feed consumption ratio (FCR) were calculated. Blood was collected at d1, 8h post-challenge, d14, and d21 of age, and serum was analyzed by ELISA for anti-OMPs specific IgY antibodies. At d7 of age, all the birds were orally inoculated with live SE (1 × 10^9^ CFU/bird). Cloacal swabs and bile samples were collected at d14 and d21 of age for anti-OMPs specific IgA antibodies analysis. At d14 and d21 of age, spleen samples were analyzed for CD4^+^/CD8^+^ T cells by flow cytometry. At d14 and d21 of age, the birds’ ceca were collected for SE quantification by plating. At d21 of age, cecal tonsils were collected to analyze inflammatory cytokine (IL-1β), anti-inflammatory cytokine (IL-10), and induced nitric oxide synthase (iNOS) mRNA amounts by RT-PCR.

### Anti-SE OMPs specific IgY and IgA antibodies in serum, cloacal swabs, and bile of chickens vaccinated *in-ovo* with CNP vaccine

Serum, cloacal swabs, and bile were collected from one bird/pen (n = 6) at d1, d7, d14, and d21 of age. The amounts of antigen-specific antibodies in serum, cloacal swab, and bile samples were determined by the enzyme-linked immunosorbent assay (ELISA), and was carried out as described previously [13]. For ELISA analysis, both treatment groups consisted of six samples, in duplicates, for each time point. Briefly, native SE OMPs were coated with either 2 μg/ml (IgY) or 7.5 μg/ml (IgA) on ELISA plates (Nunc MaxisorpTM, ThermoFisher Scientific, Waltham, MA). For analysis, 50 μl of serum and bile samples were diluted in 2.5% non-fat dry milk, and 50 μl of cloacal supernatants were added to the wells in duplicates. HRP-conjugated goat anti-chicken IgG (Southern Biotech, AL) (1: 10,000) or HRP-conjugated goat anti-chicken IgA (Gallus immunotech, NC) (1: 3000) in 2.5% skim milk powder in PBS- Tween 20 (PBST) were used as secondary antibodies. Optical density was measured as absorbance at 450 nm using a spectrophotometer (Biochek, Scarborough, ME) and values are reported as OD_450_.

### CD4^+^ and CD8^+^ cell ratios in cecal tonsils of chickens vaccinated *in-ovo* with CNP vaccine

Cecal tonsil samples were collected from one bird/pen (n = 6) at d14 and d21 of age. For flow cytometry analysis, both treatment groups consisted of six samples, in duplicates, for each time point. Cecal tonsil samples were teased over a 0.4 μm cell strainer (Sigma, MO) with 2 mL RPMI-1640 media to obtain a single-cell suspension. Single-cell suspensions were concentrated for lymphocytes by density centrifugation over Histopaque (1.077 g/mL; Sigma, MO). For CD4^+^/CD8^+^ analysis, single-cell suspensions of the cecal tonsils (1 × 10^6^ cells) were incubated with FITC-conjugated mouse anti-chicken CD4, PE-conjugated mouse anti-chicken CD8 (Southern Biotech, Birmingham, AL) at 1:200 dilution, and unlabeled mouse IgG at 1:200 dilution in a 96-well plate for 20 minutes. After incubation, cells were washed twice to remove unbound primary antibodies at 400 ×g for 5 minutes using wash buffer (1× PBS, 2 mM EDTA, 1.5% FBS). After washing, cells were analyzed using cytosoft software (Guava Easycyte, Millipore, Billerica, MA). The CD4^+^ and CD8^+^ and CD4^+^/CD8^+^ cell percentages were analyzed after gating cells based on forward-scatter and side-scatter plot for lymphocytes.

### *Salmonella* loads in the ceca of chickens vaccinated *in-ovo* with CNP vaccine

Ceca samples were collected from three birds from each pen (n = 6) at d14, and d21 of age, and samples were pulled and analyzed for SE loads by plating. Ceca samples were aseptically collected into stomacher bags, placed on ice, and transported to the laboratory. Ceca samples were macerated using a rubber mallet, and 3× (wt/vol) buffered peptone water (BPW) was added and stomached for 60 seconds and incubated for 12 h at 41°C for initial enrichment of the bacteria. A volume of 100 μL of ceca was serially diluted into 900 μL of BPW. From every dilution, a volume of 10 μL was plated in duplicates on Xylose Lactose Tergitol™ 4 (XLT4) agar plates. Plates were then incubated for 24 h at 42°C for confirmation of black colonies. Colonies were further confirmed using SYBR green qPCR with SE primers 5’-GCAGCGGTTACTATTGCAGC-3’ and 5’-CTGTGACAGGGACATTTAGCG-3’ [19]. *Salmonella* enumeration data were recorded as CFU/g of ceca and then transformed to Log 10 CFU/g of ceca for statistical analysis.

### IL-1β, IL-10 and iNOS gene expression in the cecal tonsils of chickens vaccinated *in-ovo* with CNP vaccine

Cecal tonsil samples were collected from one bird/pen (n = 6) at d21 of age. For gene expression analysis, both treatment groups consisted of six samples, in duplicates, for each time point. Total RNA was extracted using TRIzol reagent (Invitrogen) following the manufacturer’s instructions. The isolated RNA was dissolved in Tris-EDTA (pH 7.5) buffer, and the concentration was determined by using NanoDrop™ 2000c Spectrophotometer (Thermo Fisher Scientific). The cDNA synthesis was performed with 2 μg of total RNA. The mRNA transcripts analyzed for the pro-inflammatory cytokine IL-1β, anti-inflammatory cytokine IL-10, and iNOS by RT-PCR (CFX96 Touch Real-Time System, BioRad) using iQ™ SYBR® Green Supermix (Bio-Rad, CA) after normalizing for housekeeping gene β-actin mRNA. Fold change from the reference was calculated, as explained previously [20]. The primers sequences used for RT-PCR analysis in this study are described in Table 1.

**Table 1:** Primers and PCR conditions for RT-PCR.

Target Gene	Sequence	Annealing Temperature	Reference
IL-10-F	5′-CATGCTGCTGGGCCTGAA-3′	57.5°C	[21]
IL-10-R	3′ -CGTCTCCTTGATCTGCTTGATG-5′		
IL-1β -F	5′-TCCTCCAGCCAGAAAGTGA-3′	57.0°C	[22]
IL-1β -R	5′-CAGGCGGTAGAAGATGAAGC-3′		
*β-*actin-F	5′-ACCGGACTGTTACCAACACC-3′	57.0°C	[23]
*β-*actin-R	3′ -GACTGCTGCTGACACCTTCA-5′		

### Statistical analysis

For this experiment, the experimental unit was the pen, n = 6 pen/treatment, with 11 technical replicates as birds/pen. Serum, cloacal swabs, bile, and cecal tonsils samples were taken from 1 bird/pen at each time point, and samples were analyzed in duplicates. Ceca samples were taken from 3 bird/pen at each time point, and samples were pulled for each time point, and were analyzed in duplicates. All statistical differences between the two experimental treatments were determined using a parametric Student T-test. All statistical analyzes were performed using JMP Pro 14 (SAS Institute Inc., USA; 2018) with P-values <0.05 considered to be statistically significant.

## Results

### Hatchability and production performance of chickens vaccinated *in-ovo* with CNP vaccine

The hatchability of the eggs in the control group was 94%. There were no losses in the hatchability of the birds immunized with the *in-ovo Salmonella* CNP vaccine. Sixty-four out of the 68 eggs hatched in the group injected with PBS, while 68 out of the 68 eggs hatched in the group injected with the *Salmonella* CNP vaccine.

There were no significant differences (P>0.05) in the mean BWG and FCR of birds in any of the treatment groups at all-time points (Table 2).

**Table 2:** Production performance of chickens vaccinated with *in-ovo* CNP vaccine.

Treatment	0 to 21						% mortality
	Feed intake (g)	SEM	BWG (g)	SEM	FCR	SEM	
Control	900.13	23.2	697	17.3	1.29	0.04	0
CNP	996.53	17.3	730	10.0	1.37	0.03	0
P-value	0.05		0.12		0.17		

The chitosan-based *Salmonella* CNP vaccine was synthesized with SE OMPs and flagellin proteins. At embryonic d18, broiler eggs were injected with 200μl of PBS or 1000μg CNP into the amniotic cavity. At d7, birds were orally challenged with 10^9^ CFU/bird of SE. Production performance of the birds was monitored weekly and all birds were euthanized at 3 wk of age (d21). Data represents 6 pens/treatment (11 birds/pen). BWG: Body weight gain (g); FCR: Feed conversion ratio; Control: PBS-mock vaccination; CNP: chitosan nanoparticle vaccination. SEM: Standard error of mean. Data analyzed by parametric Student t-test.

### Anti-SE OMPs specific IgY and IgA antibodies in serum, cloacal swabs, and bile of chickens vaccinated *in-ovo* with CNP vaccine

**Fig 1:**
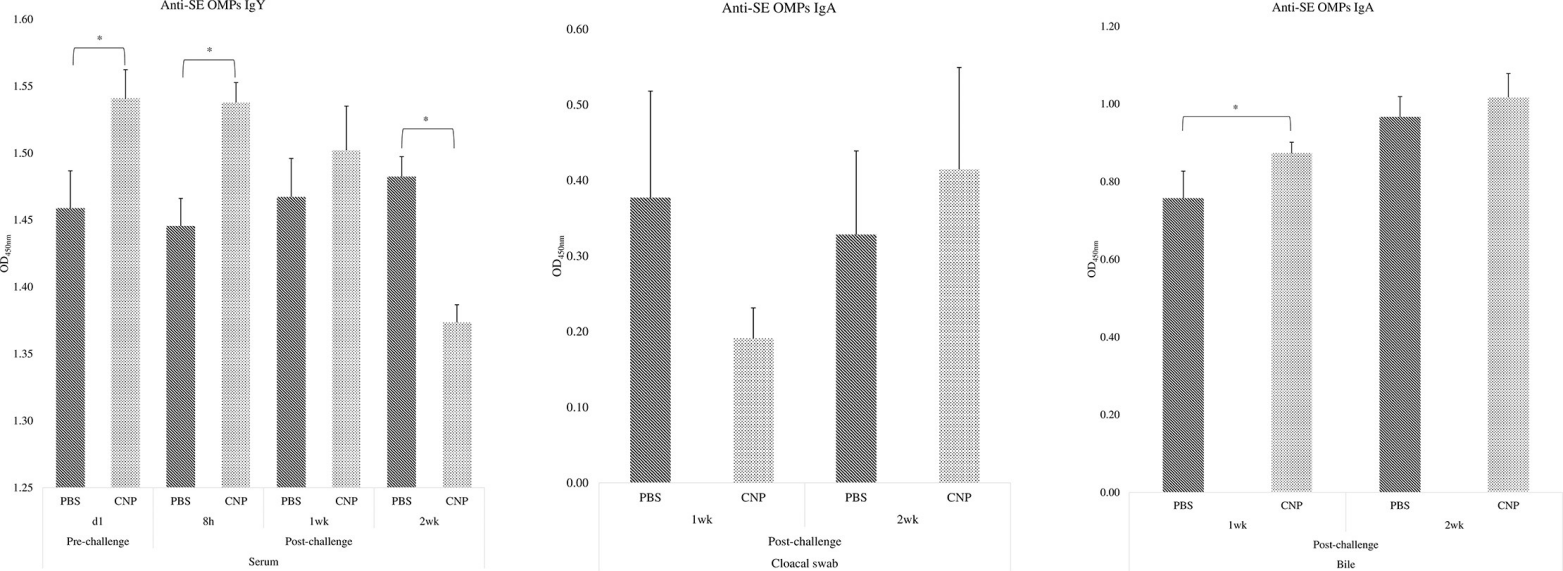
Anti-SE OMPS IgY/IgA in serum, cloacal swab, and bile of chickens vaccinated with *in-ovo* CNP. At embryonic d18, broiler eggs were vaccinated with either PBS (control) or 1000 μg CNP vaccine. At d7 of age, birds were challenged with live SE (1.0 × 10^9^ CFU/bird). Blood, cloacal swabs, and bile samples were collected pre- and post-challenge and analyzed for anti-*Salmonella* antigen-specific IgY and IgA levels by ELISA. Results were reported as average optical density (OD) values. A–Serum OMPs IgY; B–Cloacal swab OMPs IgA; C–Bile OMPs IgA. Data analyzed by parametric Student t-test; Means+SEM.Bars. n = 6. "*" means P<0.05.

At d1, birds immunized with the *in-ovo* CNP had 5.62% higher levels (P<0.05) of serum anti-SE OMPs-specific IgY, compared to control (Fig 1A). At 8h post-challenge, birds immunized with the *in-ovo* CNP had 6.39% higher levels (P<0.05) of serum anti-SE OMPs-specific IgY, compared to control (Fig 1A). At 1wk post-challenge, birds in both treatment groups had no significant differences (P>0.05) in serum anti-SE OMPs-specific IgY (Fig 1A). At 2wk post-challenge, birds immunized with the *in-ovo* CNP had 7.34% lower (P<0.05) levels of serum anti-SE OMPs-specific IgY levels, compared to control (Fig 1A).

At 1wk and 2wk post-challenge, birds in both treatment groups had no significant differences (P>0.05) of cloacal swab anti-SE OMPs-specific IgA (Fig 1B).

At 1wk post-challenge, birds immunized with the *in-ovo* CNP had 15.30% higher levels (P<0.05) of bile anti-SE OMPs-specific IgA, compared to control (Fig 1C). At 2wk post-challenge, birds in both treatment groups had no significant differences (P>0.05) of cloacal swab anti-SE OMPs-specific IgA (Fig 1C).

### CD4^+^ and CD8^+^ cell ratios in cecal tonsils of chickens vaccinated *in-ovo* with CNP vaccine

**Fig 2:**
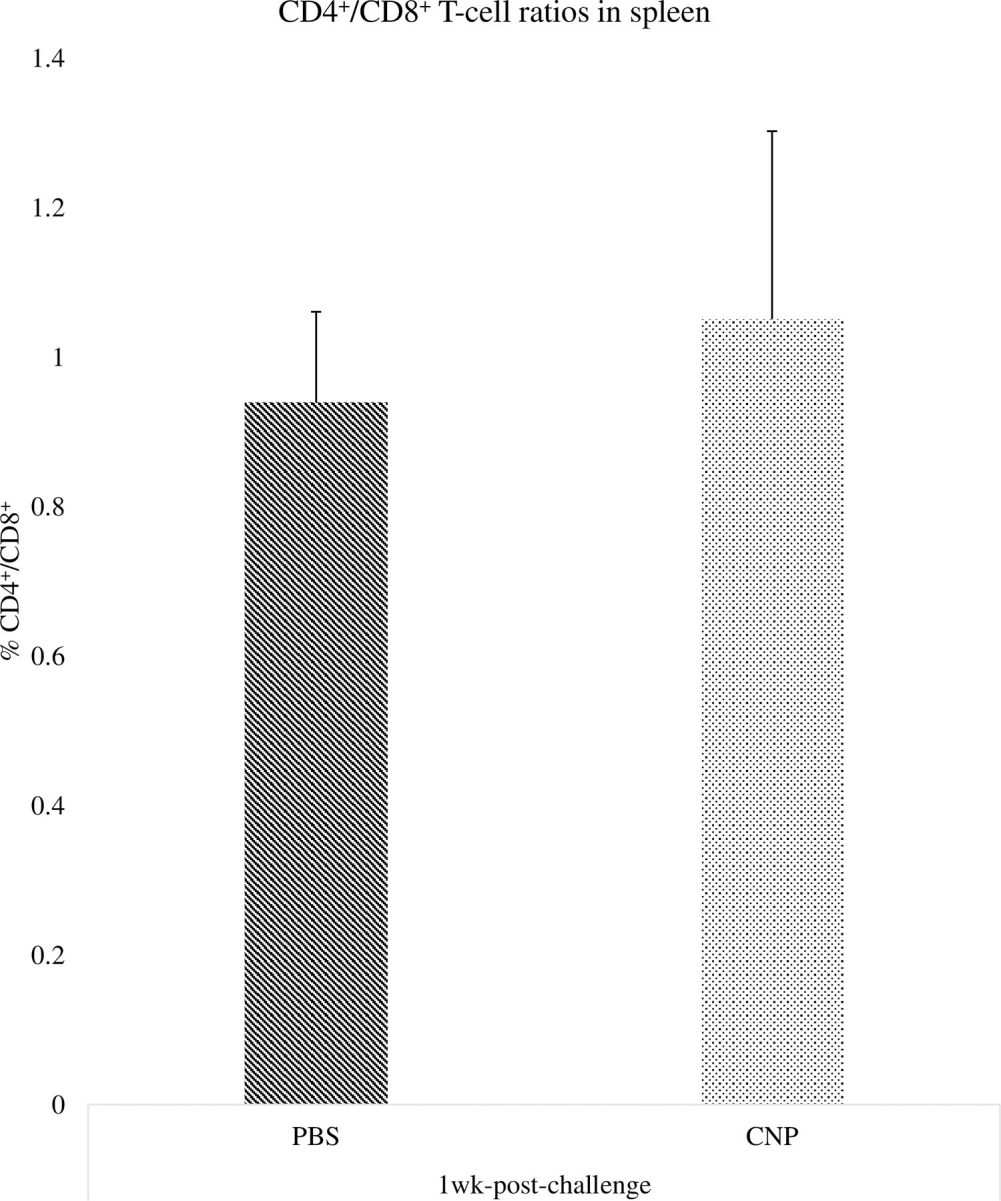
CD4^+^/CD8^+^ cell percentages in cecal tonsils of chickens vaccinated with *in-ovo* CNP at 1wk-post-challenge. At embryonic d18, broiler eggs were vaccinated with either PBS (control) or 1000 μg CNP vaccine. At d7 of age, birds were challenged with live SE (1.0 × 10^9^ CFU/bird). The CD4^+^/CD8^+^ T-cell percentage in cecal tonsils were analyzed by Flow Cytometry (Guava Eascyte; Millipore). The CD4^+^/CD8^+^ cell percentage was analyzed after gating cells based on forward-scatter and side-scatter plot for lymphocytes. Data analyzed by parametric Student t-test; Means+SEM.Bars. n = 6. "*" means P<0.05.

**Fig 3:**
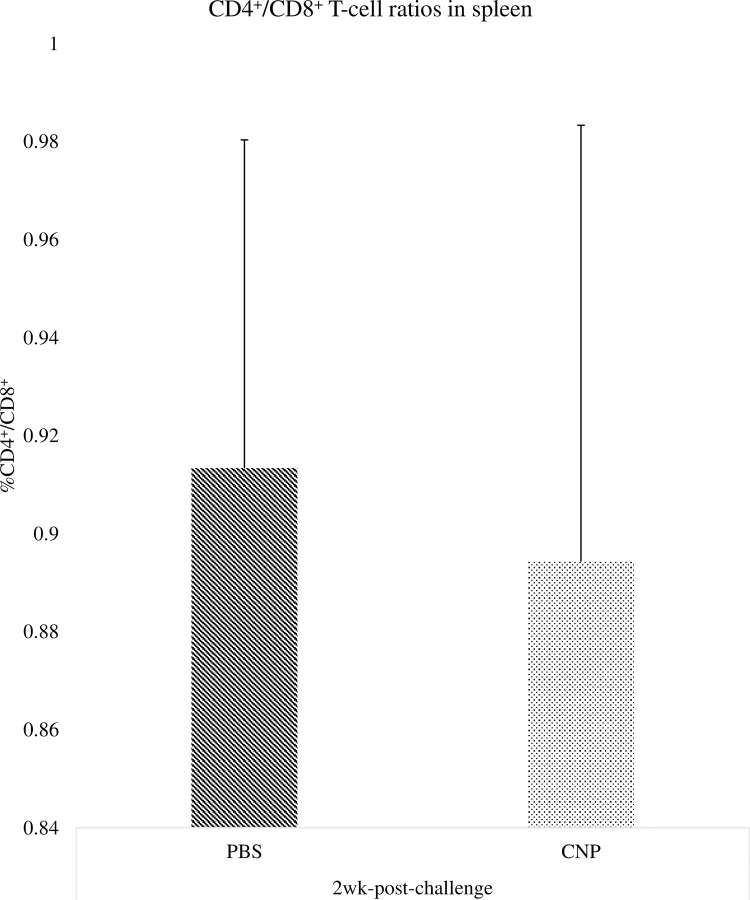
CD4^+^/CD8^+^ cell ratios in cecal tonsils of chickens vaccinated with *in-ovo* CNP at 2wk-post-challenge. At embryonic d18, broiler eggs were vaccinated with either PBS (control) or 1000 μg CNP vaccine. At d7 of age, birds were challenged with live SE (1.0 × 10^9^ CFU/bird). The CD4^+^/CD8^+^ T-cell percentage in cecal tonsils were analyzed by flow cytometry (Guava Eascyte; Millipore). The CD4^+^/CD8^+^ cell percentage was analyzed after gating cells based on forward-scatter and side-scatter plot for lymphocytes. Data analyzed by parametric Student t-test; Means+SEM.Bars. n = 6. "*" means P<0.05.

There were no significant differences (P>0.05) in the percentage of CD4^+^/CD8^+^ T-cells in cecal tonsils of birds at 1wk (Fig 2) and 2wk (Fig 3) post-challenge, compared to control.

### *Salmonella* loads in the ceca of chickens vaccinated *in-ovo* with CNP vaccine

**Fig 4:**
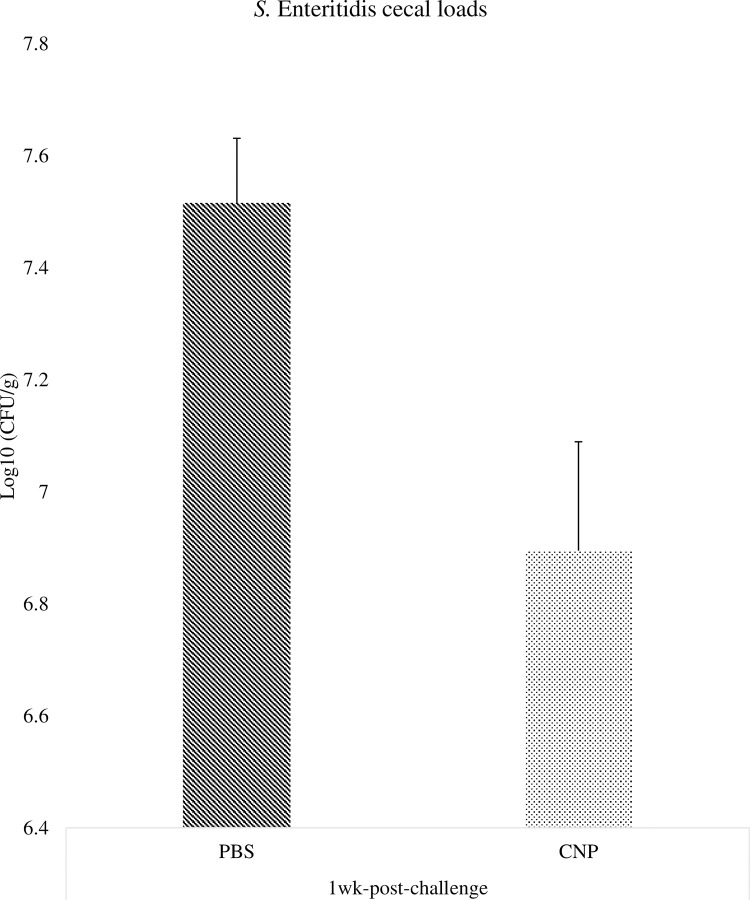
*Salmonella* loads in the ceca of chickens vaccinated *in-ovo* with CNP at 1wk-post-challenge. At embryonic d18, broilers egg were vaccinated with either PBS (control) or 1000 μg CNP vaccine. At d7 of age, birds were challenged with live SE (1.0 × 10^9^ CFU/bird). Ceca samples were collected from 3 birds/pen, pulled, and stomached for enumeration by plating on XLT-4 agar. *Salmonella* enumeration data were recorded as CFU/g of ceca and then transformed to Log 10 CFU/g of ceca for statistical analysis. Data analyzed by parametric Student t-test; Means+SEM.Bars. n = 6. "*" means P<0.05.

**Fig 5:**
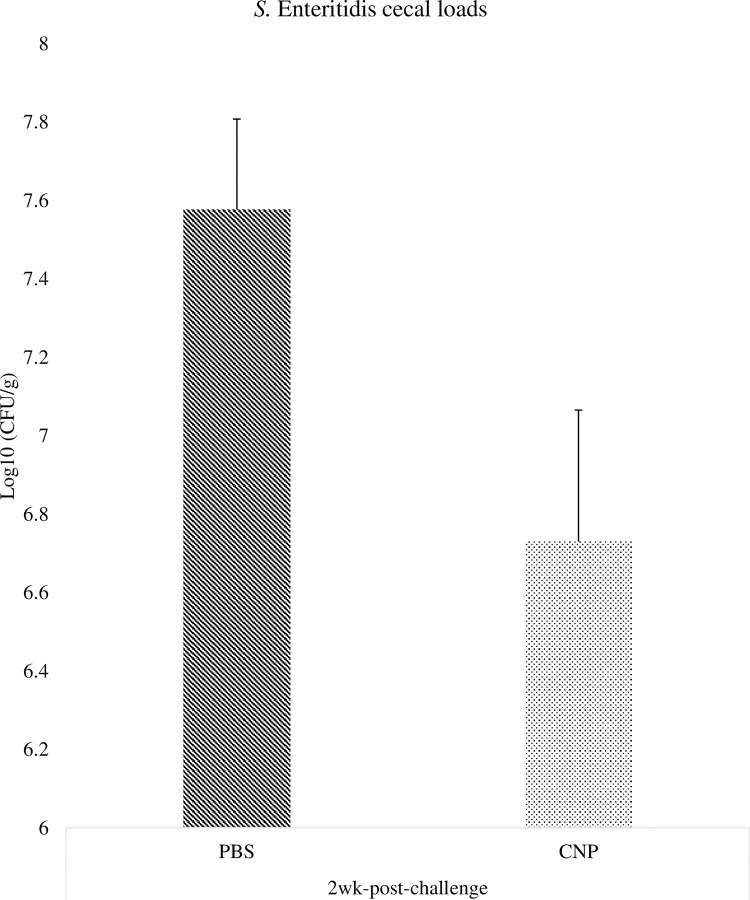
*Salmonella* loads in the ceca of chickens vaccinated *in-ovo* with CNP at 2wk-post-challenge. At embryonic d18, broiler eggs were vaccinated with either PBS (control) or 1000 μg CNP vaccine. At d7 of age, birds were challenged with live SE (1.0 × 10^9^ CFU/bird). Ceca samples were collected from 3 birds/pen, pulled, and stomached for enumeration by plating on XLT-4 agar. *Salmonella* enumeration data were recorded as CFU/g of ceca and then transformed to Log 10 CFU/g of ceca for statistical analysis. Data analyzed by parametric Student t-test; Means+SEM.Bars. n = 6. "*" means P<0.05.

At 1wk post-challenge, birds immunized with the *in-ovo* CNP vaccine had a 0.62 Log 10 CFU/g reduction (P<0.05) of SE population in the ceca, compared to control (Fig 4). At 2wk post-challenge, birds immunized with the *in-ovo* CNP vaccine had a 0.85 Log 10 CFU/g reduction (P<0.05) of SE population in the ceca, compared to control (Fig 5).

### Cytokine gene expression in the cecal tonsils of chickens vaccinated *in-ovo* with CNP vaccine

**Fig 6:**
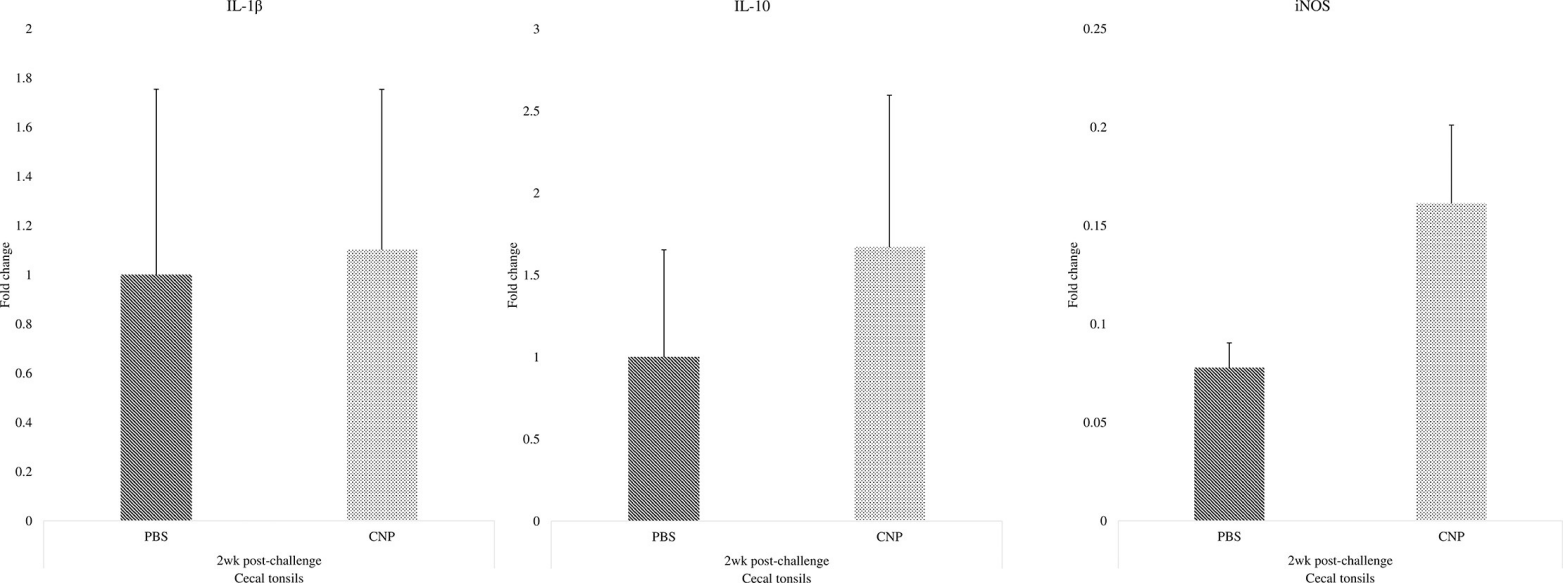
Cytokine gene expression in the cecal tonsils of chickens vaccinated *in-ovo* with CNP. At embryonic d18, broiler eggs were vaccinated with either PBS (control) or 1000 μg CNP vaccine. At d7 of age, birds were challenged with live SE (1.0 × 10^9^ CFU/bird). Cecal tonsil samples were collected at 2wk post-challenge and analyzed for cytokine mRNA amounts by RT-PCR. Data represented fold change compared to control. A–IL-1β mRNA; B–IL-10 mRNA; C–iNOS mRNA. Data analyzed by parametric Student t-test; Means+SEM.Bars. n = 6. "*" means P<0.05.

There were no significant differences (P>0.05) in the IL-1β (Fig 6A), IL-10 (Fig 6B), or iNOS (Fig 6C) mRNA amounts at 2wk post-challenge when compared to control.

## Discussion

The physicochemical properties of the CNPs were determined in a previous study [12]. The average size of the nanoparticles was 168 nm, the entrapment efficiency of the nanoparticles was 70%, and the surface labeling efficiency of flagella on chitosan nanoparticles was 25%. The chitosan nanoparticles were found to be stable in both acidic and alkaline pH conditions with less than 10% and 0% protein release. The biocompatibility of chitosan nanoparticles using a hemolysis assay resulted in 0% hemolysis, demonstrating that the nanoparticles were biocompatible in chickens. Moreover, a previous study with empty CNPs alone showed they did not improve antibody response in the absence of a *Salmonella* antigen [12].

The findings from this study suggest that the novel CNP oral vaccine administered *in-ovo* for *Salmonella* control in broilers is a promising candidate vaccine against *Salmonella*. The mass administration of the *Salmonella* CNP vaccine ultimately induced an anti-*Salmonella* antigen-specific systemic and mucosal immune response and decreased SE cecal colonization in broilers.

A successful *in-ovo* vaccination should not decrease hatchability [24]. The limitations of the present study was the *in-ovo* vaccine was injected manually inside the embryo. Even with a manual *in-ovo* injection, a 97% hatchability was observed. The efficacy of both manual and automatic injections has been demonstrated [24]. Nevertheless, commercial application mandates automation, which can vaccinate a larger number of eggs efficiently, and reduce labor costs and the chances of human error. *In-ovo* vaccination is currently the standard procedure for Marek ’s disease and infectious bursal disease vaccines applied in hatcheries in the USA. Future studies should incorporate automation for the *in-ovo* vaccination of the *Salmonella* CNP vaccine as the vaccine has shown to have protective effects against SE and Heidelberg serovars [13].

There was no significant effect of the *Salmonella* CNP vaccine on the bird’s production performance. The results demonstrate that the embryonic vaccination had no negative effects on the production performance of broiler birds. The absence of difference in the performance can be seen as a positive result as the vaccine is protective against SE and does not significantly compromise the bird’s health. Previous studies have shown that chitosan nanoparticles are safe in chicken [12, 25, 26]. Other studies have shown that gavaging chickens with chitosan nanoparticles had no effect on mortality [25, 27]. Similarly, no mortality was observed during the 21-day experimental period. Our results demonstrated that the *in-ovo* administration of the *Salmonella* CNP candidate vaccine was safe with no adverse effects on bird performance.

The OMPs-specific IgY and IgA antibody responses were analyzed in serum, cloacal swabs, and bile samples. The *in-ovo* administration of the CNP vaccine induced antigen-specific antibody protection against SE at day-of-hatch and can potentially protect against SE infection as early as 8h post-challenge. Significant higher IgY antibody levels were also observed at 1wk post-challenge while at 2wk post-challenge the control animals displayed a significantly higher concentration of antibodies against SE in the serum. The increasing antibody levels can be attributed to a persistent infection in a carrier animal [28]. This may be because the non-vaccinated group had 0.62 Log 10 CFU/g more *Salmonella* load at 1wk post-challenge and hence can be expected to be in the process of eliminating the pathogen while the CNP vaccinated group had already cleared the infection by 0.85 Log 10 CFU/g at 2wk post-challenge, which is consistent with the CD4^+^/CD8^+^ cells percentage results obtained in this study.

Secretory IgA is the most abundant antibody class in intestinal secretions [29], and it serves as the first line of defense against enteric pathogens, such as *Salmonella*. Significantly higher bile anti-SE OMPs IgA levels were observed at 1wk post-challenge in birds vaccinated with CNP compared to that in the control group. The vaccine also increased antigen-specific IgA at 2wk post-challenge in bile and cloacal swabs. Our findings demonstrate that the *in-ovo* administration of the candidate vaccine can elicit a *Salmonella*-specific mucosal IgA response. Results are in agreement with previous findings, as the vaccine under study has shown to induce an antigen-specific mucosal IgA response in layers and broilers challenged with *Salmonella* [12, 13].

Our ELISA results demonstrated that the *in-ovo* administration of the *Salmonella* CNP vaccine can induce both antigen-specific mucosal and systemic immune responses against SE.

Interestingly, our findings indicate that the *in-ovo* CNP vaccinated group had a numerical increase in the frequency of CD4^+^ T-cells at 1wk post-challenge when compared to control. The immune response of *Salmonella*-specific CD4^+^ T-cells suggests a way for the host to quickly produce interferon-γ to activate macrophages and protect against *Salmonella* [29]. Nevertheless, is important to recall that despite the rapid development of a large number of CD4^+^ T-cells during primary *Salmonella* infection, there is actually very little evidence to suggest that, at this early stage of infection, they contribute to bacterial clearance [30]. Mostly because at an early phase of *Salmonella* infection, *Salmonella* can survive and replicate within macrophages [30]. At 2wk post-challenge, the PBS (control) group had higher CD4^+^ T-cells frequency, indicating that the non-vaccinated group could still be trying to eliminate the infection at 2 wk post-challenge, as supported by our serology findings, whilst the CNP vaccinated birds have cleared the SE infection.

Birds can remain asymptomatic with up to Log 5 of *Salmonella* CFU [1]. This resistance is thought due to the activation of T-regulatory cells and their suppressive immune response by IL-10 [31]. For this experiment, both treatment groups were kept under the same experimental conditions; thus, all birds were challenged with SE. The SE load in ceca of birds immunized with the *in-ovo* CNP vaccine was reduced compared to the control group. Reduction in SE intestinal loads is critical to prevent the transfer of *Salmonella* from poultry to humans. A successful *Salmonella* vaccine in the poultry industry should ideally reduce the total intestinal bacterial loads and shed in feces; however, this has yet not been feasible. As an alternative, an efficient vaccine that can reduce *Salmonella* loads is combined with other on-farm control strategies, to ultimately decrease foodborne illness risks for humans [32]. Findings demonstrate that the CNP vaccine is a promising candidate to mitigate *Salmonella* in broilers, as its *in-ovo* administration has shown to decrease SE cecal colonization.

*In-ovo* immunization with CNP *Salmonella* vaccine had no negative effects on anti-inflammatory IL-10 or pro-inflammatory IL-1β mRNA expression in cecal tonsils. However, there was an increase in both IL-10 and IL-1β mRNA expression in caecal tonsils of CNP vaccinated broilers, compared to control group. This is consistent with what has been found in previous research with chitosan nanoparticle vaccines [12, 13]. The oral gavage of the CNP *Salmonella* vaccine has shown to induce balanced levels of IL-1β and IL-10 cytokines in layers and broilers challenged with SE. The higher IL-1β levels are attributed to the intrinsic adjuvant composition of the vaccine that can induce a predominantly Th1-type inflammatory response [33]. The anti-inflammatory IL-10 cytokine may be increased to reduce the host tissue damage in response to the inflammation caused by *Salmonella* infections [34].

*In-ovo* immunization with CNP *Salmonella* vaccine had no negative effects on iNOS mRNA expression in cecal tonsils. However, there was a numerical increase in iNOS mRNA expression in caecal tonsils of CNP vaccinated broilers, compared to the control group. Results are in agreement with previous findings, as the oral gavage of the CNP *Salmonella* vaccine has been shown to induce higher levels of iNOS in broilers challenged with SE [13]. The iNOS is a key enzyme in the macrophage inflammatory response, as it is the source of nitric oxide (NO) that is potently induced in response to pro-inflammatory stimuli and will function to kill pathogens like *Salmonella* [35]. However, NO production can decrease the production performance of broilers [36], which was not the case in this study, and remains consistent with our previous findings [13]. Overall, the *in-ovo* administration of the CNP vaccine had no negative effects on the birds’ immune status.

## Conclusion

The oral gavage of the CNP *Salmonella* vaccine has previously been shown to induce significantly higher antigen-specific IgA response in bile, serum, cloacal swab, and tracheal wash samples of layers challenged with *Salmonella* [12]. The oral gavage of 1000μg CNP *Salmonella* vaccine has previously been shown to induce significantly higher antigen-specific IgY and IgA antibodies in serum, cloacal swab, and bile samples of broilers challenged with *Salmonella* [13]. The results of the present study showed that the CNP can also be mass administered by *in-ovo*, making it a potential vaccine candidate to mitigate *Salmonella* in poultry. Findings demonstrate that the *in-ovo* vaccination of the CNP *Salmonella* vaccine can induce antigen-specific systemic and mucosal immune response without causing significant hatchability losses or altering the performance of broilers. The vaccine under study demonstrated promising results on significantly reducing SE cecal colonization in birds. Also, the *in-ovo* CNP vaccine had no negative effects on the bird’s production performance, CD4^+^/CD8^+^ T-cells frequency, pro- and anti-inflammatory cytokines levels, or iNOS levels of birds. Future research will further study the vaccine’s potential for mass vaccination methods.

## Abbreviations

CNP: Chitosan nanoparticle
SE: *Salmonella* Enteritidis
OMPs: Outer membrane proteins
BWG: Body weight gain
FCR: Feed conversion ratio
